# A Network-Based Model of Oncogenic Collaboration for Prediction of Drug Sensitivity

**DOI:** 10.3389/fgene.2015.00341

**Published:** 2015-12-23

**Authors:** Ted G. Laderas, Laura M. Heiser, Kemal Sönmez

**Affiliations:** ^1^OHSU Knight Cancer Institute, Oregon Health & Science University, PortlandOR, USA; ^2^Department of Medical Informatics and Clinical Epidemiology, Oregon Health & Science University, PortlandOR, USA; ^3^Department of Biomedical Engineering, Knight Cancer Institute, Oregon Health & Science University, PortlandOR, USA

**Keywords:** network, oncogenic collaboration, breast neoplasms, drug sensitivity, survival

## Abstract

Tumorigenesis is a multi-step process, involving the acquisition of multiple oncogenic mutations that transform cells, resulting in systemic dysregulation that enables proliferation, invasion, and other cancer hallmarks. The goal of precision medicine is to identify therapeutically-actionable mutations from large-scale omic datasets. However, the multiplicity of oncogenes required for transformation, known as oncogenic collaboration, makes assigning effective treatments difficult. Motivated by this observation, we propose a new type of oncogenic collaboration where mutations in genes that interact with an oncogene may contribute to the oncogene’s deleterious potential, a new genomic feature that we term “surrogate oncogenes.” Surrogate oncogenes are representatives of these mutated subnetworks that interact with oncogenes. By mapping mutations to a protein–protein interaction network, we determine the significance of the observed distribution using permutation-based methods. For a panel of 38 breast cancer cell lines, we identified a significant number of surrogate oncogenes in known oncogenes such as BRCA1 and ESR1, lending credence to this approach. In addition, using Random Forest Classifiers, we show that these significant surrogate oncogenes predict drug sensitivity for 74 drugs in the breast cancer cell lines with a mean error rate of 30.9%. Additionally, we show that surrogate oncogenes are predictive of survival in patients. The surrogate oncogene framework incorporates unique or rare mutations from a single sample, and therefore has the potential to integrate patient-unique mutations into drug sensitivity predictions, suggesting a new direction in precision medicine and drug development. Additionally, we show the prevalence of significant surrogate oncogenes in multiple cancers from The Cancer Genome Atlas, suggesting that surrogate oncogenes may be a useful genomic feature for guiding pancancer analyses and assigning therapies across many tissue types.

## Introduction

In oncogenic collaboration, multiple cellular systems are dysregulated as key hallmarks in tumorigenesis, reflected in multiple mutations targeting multiple cellular systems (Hanahan and Weinberg, 2000, 2011). Given this oncogenic collaboration, a key problem is prioritizing targeted therapies in individuals, which is compounded by the lack of highly prevalent oncogenes in patient populations. Even well known driver oncogenes such as BRCA1 and BRCA2 have a relatively low prevalence (∼12%) in breast cancer populations (Cancer Genome Atlas Network, 2012). These rare or infrequent mutations comprise the majority of mutations in cancer populations, and are known as the ‘long-tail’ of mutations (Looking Across Many Cancer Genomes, 2013). Unfortunately, most statistical methods, such as MutSigCV and MuSiC (Dees et al., 2012; Lawrence et al., 2013), are frequency-based and do not incorporate these rare mutations, often assigning them as “passenger,” or non-driver mutations due to rarity in the patient population.

**FIGURE 1:**
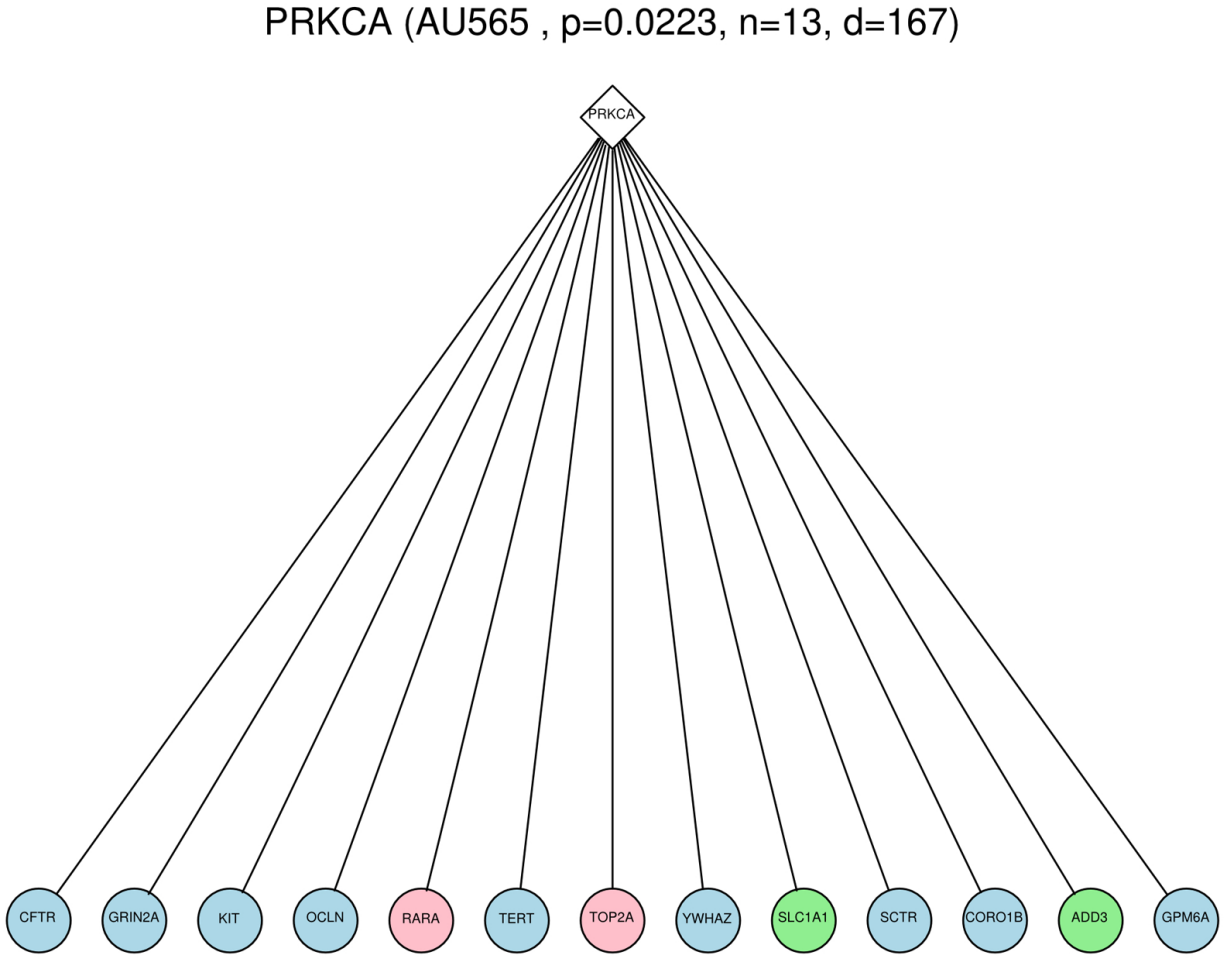
**Example of a Surrogate oncogene, PRKCA, observed within the AU565 cell line.** The diamond node is the surrogate oncogene itself, and the circles are immediate neighbors in the PPI network that show genomic alterations (light blue = mutation, pink = copy number gain, green = copy number loss). The number of mutated neighbors (13) was shown to be significant (*p*-value = 0.0223) compared to the count of 10000 permutations of a randomly mutated network.

We postulate that the long-tail mutations themselves have network effects by influencing interactions with neighbors in the protein network. By surveying a set of oncogenes and their immediate neighbors on a protein–protein interaction (PPI) network, we show that within a sample, neighboring mutations cluster around known oncogenes. We summarize these mutational clusters/subnetworks as a single ‘surrogate oncogene’ and suggest that they may cooperate toward dysregulation of the oncogene (**Figure 1**). Additionally, using surrogates, we account for oncogenic collaboration and show that surrogate oncogenes predict drug sensitivity in cell lines with accuracy equal to features that predict subtype.

## Background

### Network Approaches to Integrating Mutation and Copy Number Alteration (CNA) Data

A number of network-based approaches for assessing the functional impact of mutations have been used to analyze cancer genomic data (for a summary see Gulati et al., 2013). These network-based approaches essentially search for oncogenic collaboration by highlighting important interactions within the network of interest. Most approaches utilize a PPI network such as the Human Protein Reference Database (HPRD), or STRING, although there are several that use transcriptional networks. By annotating mutations on these networks, the network-based methods aim to ascribe certain properties to these mutations, such as connectivity or path distance to key signaling proteins. We have examined four network-based approaches for integrating these data: MEMo (Ciriello et al., 2011, 2013), HotNets (Vandin et al., 2012), DriverNet (Bashashati et al., 2012), and network based stratification (NBS; Hofree et al., 2013) (**Table 1**). The algorithms vary with respect to five aspects: (1) search strategy, (2) networks used, (3) statistical framework, (4) whether subtypes can be defined by the algorithm, and (5) whether the output can be individualized on a per-sample level.

**Table 1:** Comparison of network-based methods for integrating mutation and copy number alteration (CNA) data.

Method	MeMO (Ciriello et al., 2011, 2013)	DriverNet (Bashashati et al., 2012)	HotNets (Vandin et al., 2012)	NBS (Hofree et al., 2013)	Surrogate oncogene
**Search strategy**	Mutual exclusivity	Expression driven	Diffusion-based	Diffusion-based	Oncogene focused
**Networks used**	PPI	PPI	Pathway-based	Multiple	PPI
**Statistical testing**	Clique analysis	Bipartite graph based statistics	Permutation	None (clustering based)	Permutation
**Defined subtypes**	None	None	None	Network clustering	Survival tree
**Individualized**	No	No	No	No	Yes


**FIGURE 2:**
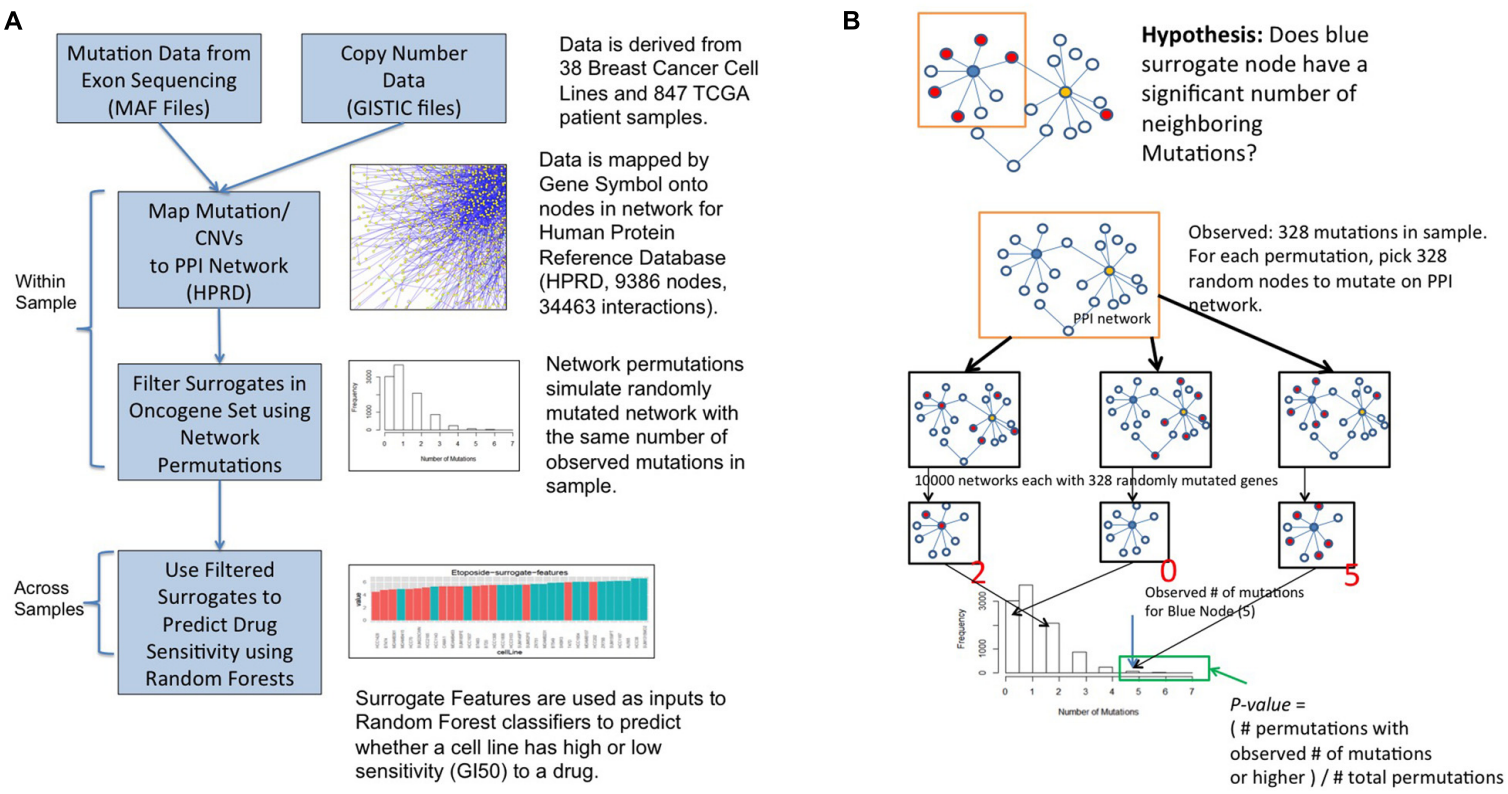
**Surrogate oncogene workflow (A) and statistical framework (B)**.

In contrast to these other approaches, we suggest a *within-individual* (that is, within a single sample) based approach to genomic alterations. We examine a set of known oncogenes in order to investigate the possible role of neighboring mutations in their regulation as possible oncogenic collaborators. We term the nodes in the subset that have a significantly higher than randomly expected number of mutated neighbors as *surrogate oncogenes*. Surrogate oncogenes are thus representatives of these mutated subnetworks. Because these nodes have high connectivity, we define a statistical background model for deciding whether the number of neighboring mutations that an oncogene has is significantly greater than expected by chance (**Figure 2B**). We suggest surrogate oncogenes as a new model of oncogenic collaboration that can indicate an oncogenic role for unique mutations previously classified as passenger mutations. Additionally, we show that surrogate oncogenes are associated with drug sensitivity, subtype, and survival, which indicates their potential for use in precision medicine applications.

## Methods

### Mutation and Copy Number Data

The breast cancer cell lines used in this study are described in Neve et al. (2006). Copy number and mutation data for the breast cancer cell lines were obtained from the DREAM 7 breast cancer drug sensitivity challenge and the Integrative Cancer Biology Program (ICBP) Data Portal (https://nciphub.org/resources/622) (Heiser et al., 2012; Daemen et al., 2013; Costello et al., 2014). Copy number data were derived from segmented Affymetrix SNP 6.0 arrays processed using the GISTIC 1.0 copy number algorithm. Only genes that were in high confidence peaks of amplifications and deletions were included. Mutations in cell lines were called through comparison with the NCBI 37 reference, and filtered by occurrence in dbSNP. Allele counts went through a mutation calling pipeline and were filtered for high base quality (≥10), high neighborhood base quality (≥10), and high mapping quality (≥20) of associated reads. The likelihoods of all possible genotypes at a site were calculated, and use as the input for a Bayesian model that incorporated the prior probability for the reference call, and incorporated the heterozygous rate of the human genome. All heterozygous or homozygous mutants alleles were then filtered by the following metrics: genotype quality (≥100), total depth (≥8), and mutant allele strand bias (*p*-value < 0.005). Additionally, all mutations were filtered by whether the SNP occurred in dbSNP, a database of SNP variants. For the TCGA patients, copy number (as GISTIC files) and somatic mutation (as MAF files) data were obtained from the PanGEA PanCancer portal^1^ in the form of GISTIC files and Mutation Annotation Format (MAF) files, with additional GISTIC information derived from the Broad Institute website.

### Workflow for Surrogate Analysis

The workflow for surrogate oncogene analysis is shown in **Figure 2A** GISTIC (Mermel et al., 2011) files are used to select genes that are amplified or deleted in each sample. Mutations for each sample are derived from the MAF files (Mutation Annotation Format (MAF) Specification, 2014). These genomic alterations are then superimposed onto a protein–protein interaction network (HPRD release 9). For a set of oncogenes of interest, termed the surrogate oncogene set (see below), we apply the surrogate analysis on a per-sample basis. Finally, statistically significant surrogate oncogenes are used as input features to a Random Forest (RF) classifier to predict whether a cell line is relatively sensitive or insensitive to a particular drug. We compare our classifier results to those of the classifier based on the genes used to determine the PAM50 molecular subtype, another molecular feature set used to predict drug sensitivity in breast cancer (Parker et al., 2009).

### Selection of Surrogate Oncogene Set

The selection of the surrogate gene set is tumor-specific and based on a network expansion approach using an initial seed set. Within breast cancer, this initial seed gene set was derived by selecting two sets of genes from the TCGA Breast Tumor paper: the most frequently mutated genes in all samples, and the most frequently mutated genes within the molecular subtypes (Cancer Genome Atlas Network, 2012). This initial seed set of 54 genes was expanded by including immediate neighbors from HPRD filtered by their connectivity to the seed set. Those neighbors with at least two connections to genes in the seed set were included, a threshold that was decided through examining the frequency distributions of number connections to the seed set (**Supplementary Figure S1**). The total number of genes in the surrogate set is 180. For the additional cancers in TCGA, we obtained similar lists of genes from other similar analyses in the scientific literature and expanded them using the same network strategy (BLCA: *n* = 88, GBM = 40). We noticed similar distributions of first connected neighbors to each seed set.

### Drug Sensitivity Data

Drug sensitivity data was from Heiser et al. (2012) in the form of Growth Inhibition at 50 percent (GI50) data, a measure of the concentration of the drug required to inhibit growth by 50 percent. For each drug, GI50 data was discretized using equiprobable binning into equal-sized bins of high and low sensitivity. This strategy was chosen to address a known bias in the RF algorithm to choose the larger group in an unbalanced design (Breiman, 2001).

### Statistical Framework for Surrogate Oncogenes

A permutation-based framework was used to determine significance of a surrogate oncogene (**Figure 2B**). Within a sample and for each gene in the surrogate set, we asked whether the number of neighboring mutations is higher than a background null distribution. The background distribution for each surrogate oncogene was derived by randomly mutating the entire PPI network with the same number of mutations as observed in the sample. A *p*-value was calculated from the proportion of permuted samples that have the observed number of neighboring mutations or higher.

### Prediction of Drug Sensitivity using Surrogate Oncogenes

Statistically significant surrogate oncogenes were then used as input features to a RF classifier, in addition to mutations within the surrogate oncogene set for each of the 74 drugs tested. We refined the classifiers by running the RF algorithm, calculating Mean Gini Importance (a purity-based metric) to rank the features, and then re-running the RF classifier with the top 10% of ranked genes. Using this procedure increased the overall accuracy of the classifier. Cross-validation error was calculated as the out-of-bag (OOB) error for the RF classifier. We performed classification based on the PAM50 gene set to assess performance of our drug sensitivity predictions. We then compared across the PAM50 expression features and the surrogate/mutated features.

### Association of Surrogate Oncogenes with Clinical Features

We also tested the association of surrogate oncogenes with two clinically relevant features: molecular subtype and survival. PAM50 molecular subtype calls were obtained for both the breast cancer cell lines and TCGA Breast Cancer patients from Synapse^2^ Statistically significant surrogate oncogenes were used in a Fisher’s test of association with molecular subtype for both the cell lines and patients. Additionally, surrogate oncogenes were considered as binary features (0 = absent, 1 = present, alpha = 0.05) and survival trees were generated using the rpart (4.1-10) package in R (R version 3.1). The initial survival tree was pruned to four nodes total using a complexity criterion and the subsequent groupings were plotted as a Kaplan–Meier survival curve.

### Surrogate Oncogene Explorer

We created a web application to visualize surrogate oncogenes in the form of an interactive heatmap^3^ Alpha, the level of significance, can be chosen in order to assess the effect on overall significance. Surrogate features and cell lines can be ordered by different qualities (e.g., number of mutations observed in a cell line, total number of connections in a surrogate) in order to assess their effect on the analysis. Individual surrogate oncogenes within a cell line can be visualized and downloaded. Mutations in the surrogate oncogenes themselves can be overlaid on the significant genes in the heatmap in order to provide additional information.

### Code Availability

The current R code requires GISTIC files and MAF files as input. The code is open-source and currently available at https://github.com/laderast/. A full description of the surrogateMutation package using the TCGA breast cancer patient data is included there.

## Results

### Surrogate Oncogenes are Highly Prevalent in Breast Cancer Cell Lines

**FIGURE 3:**
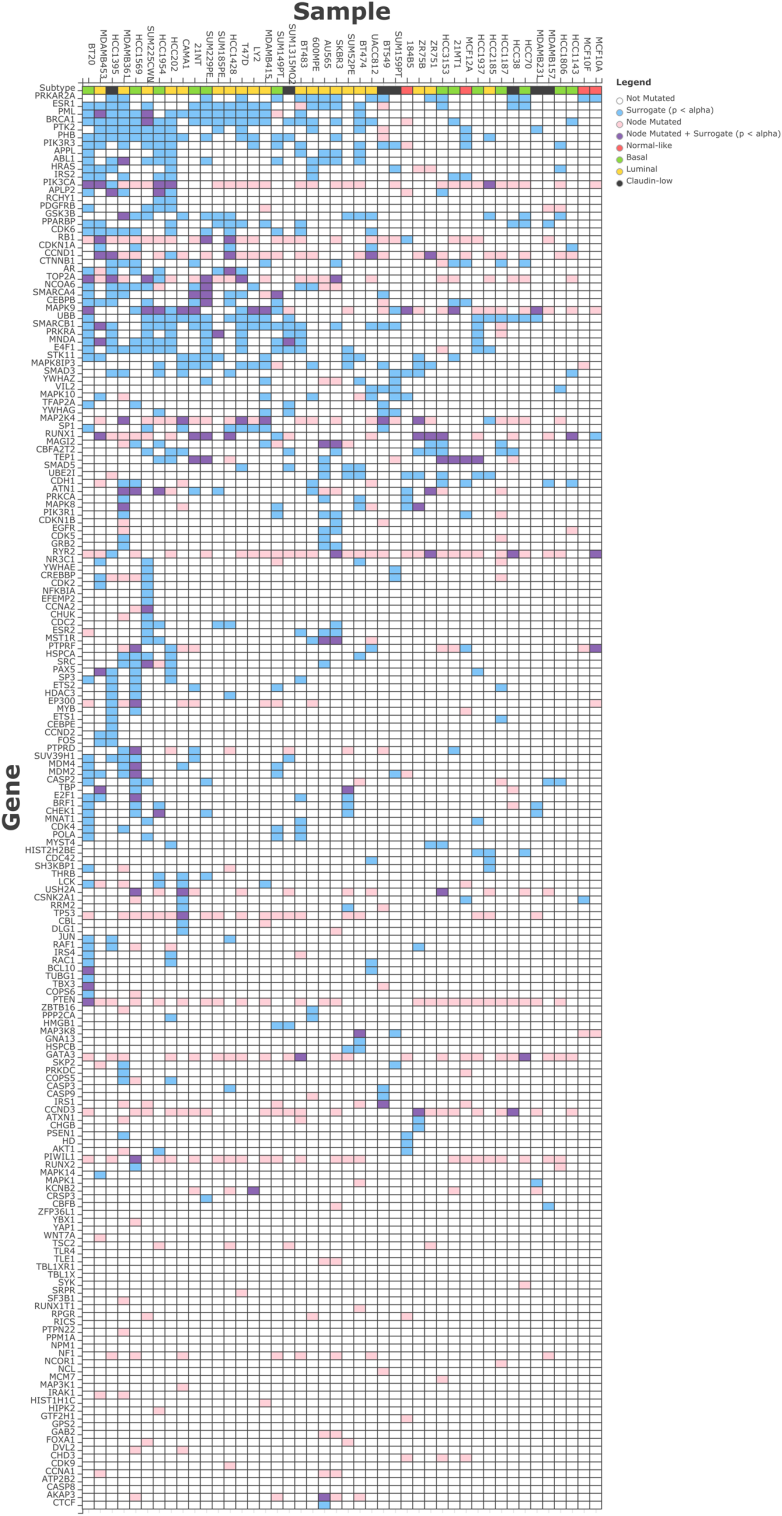
**Surrogate oncogenes within Breast Cancer Cell Lines.** The heatmap above shows significant (*p* < 0.05) surrogate oncogenes (rows) for each cell line (columns) as light blue boxes. Additionally, if a genetic alteration was observed in that oncogene, the box is colored pink, or if it has both a significant surrogate and is also altered, it is colored purple. Cell lines and surrogate oncogenes are ordered by clustering on both rows and columns of the surrogate features.

We observed an average of 17 surrogate oncogenes per sample across the breast cancer cell lines (**Figure 3**), with a range from 2 in MCF10F to 50 in BT20. Within a cell line, the number of surrogates observed is not correlated with the total number of genetic alterations in the cell line (data not shown). Similarly, across the surrogate oncogene set, the prevalence of the oncogene from a surrogate oncogene set across the cell lines is not always associated with the total degree, or connectedness of the oncogene (**Supplementary Figures S2** and **S3**). For example, BRCA1 (significant in 21/44 cell lines) has moderately high connectivity (101 neighbors) compared to the mean connectivity of all surrogate oncogenes (47 neighbors). However, BRCA1’s connectivity is not as high as TP53 (237 neighbors) and YWHAG (240 neighbors). RB1, a highly connected (123 neighbors) tumor suppressor gene essential for cell cycle progression is also significant in a large number of the cell lines.

### Surrogate Oncogenes Occur in Patient Populations

**FIGURE 4:**
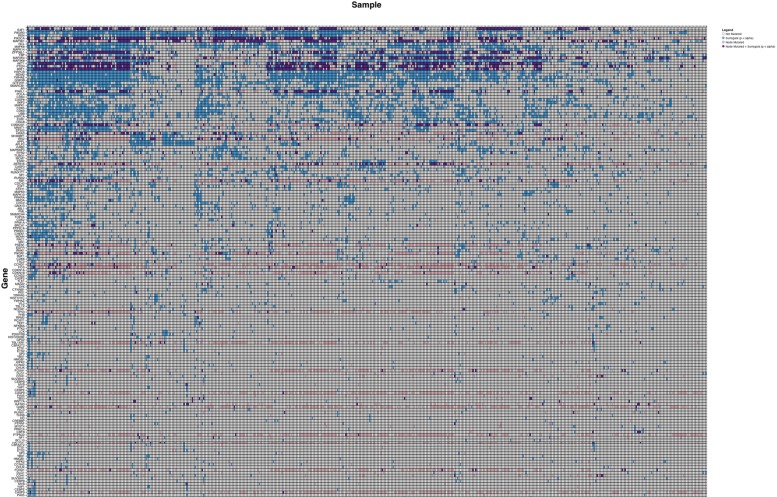
**Surrogate oncogenes in TCGA Cancer Patients.** Heatmap is ordered and annotated identically to **Figure 3**

We initially analyzed the TCGA breast cancer cohort (*n* = 487) in order to assess whether surrogate oncogenes were a generalizable phenomenon that also occur in patient samples. Surrogate oncogenes are highly prevalent within the breast tumor population, with a mean number of 21.2 significant surrogate oncogenes per tumor (*n* = 487) (**Figure 4**). As compared to the breast cancer cell lines, the number of mutated/altered neighbors is lower in patient samples. Additionally, copy number alterations comprised a larger percentage of neighbors in the patient population than in cell lines (87% and 30%, respectively). Additionally, we conducted a similar analysis for bladder cancer (BLCA, mean surrogates 3.52, *n* = 97), and glioblastoma (GBM, mean surrogates 3.12, *n* = 265) and showed that for each of these cancers, surrogate oncogenes are statistically significant across a wide variety of patients (**Supplementary Tables S1**–**S3**).

### Surrogate Oncogenes Incorporate Low Frequency or ‘Long-tail’ Mutations

By summing the frequency of neighboring mutations for a single surrogate across all patients, our analysis can incorporate rare mutations in the patient population. For BRCA1 in the cell lines (**Figure 7**), some neighbors are frequently mutated across all samples (TP53, SMAD2, and RB1), whereas others are rarely mutated (STAT1, STAT3, ABL1). Note that these infrequent mutations are highly connected to other neighbors of BRCA1, suggesting that they may have a strong influence on the BRCA1 subnetwork.

### Surrogate Oncogenes are Associated with Molecular Subtype in Breast Cancer Cell Lines and Patients

**Table 2** shows the results of a Fisher’s exact test of association between individual oncogenes from significant surrogate oncogene sets and PAM50 Molecular Subtypes for 41 of the cell lines. Of the three subtypes available in the cell line dataset (basal, claudin-low, and luminal), a number of surrogates are associated with a subtype. For example, there is significant association between BRCA1, ESR2, MAP2K4, and PML surrogate oncogenes and luminal subtype. Surrogates are also associated with PAM50 subtype calls in the TCGA Breast Cancer patients (*n* = 487, **Supplementary Table S4**). Because of the larger number of samples, a larger number of surrogates show an association with each subtype. Caution must be made in the analysis of surrogate oncogenes using gene set analysis methods, as the surrogate set is preselected from known oncogenes and is biased. Overrepresentation analysis, such as gene enrichment analysis (GSEA), assumes an unbiased set of genes and thus is not an appropriate framework for analyzing the surrogate set (Subramanian et al., 2005).

**Table 2:** Surrogate oncogenes associated with molecular subtype in breast cancer cell lines (*p*-value < 0.05).

Surrogate oncogene	Basal	Claudin-low	Luminal
BRCA1	1.000	0.232	**0.040**
ESR2	0.302	1.000	**0.036**
ETS2	**0.022**	1.000	0.053
MAP2K4	0.086	1.000	**0.035**
PML	0.740	0.220	**0.013**
TEP1	**0.012**	0.314	0.150
YWHAG	1.000	**0.023**	0.356


*p-values for Fisher’s exact test are reported for each subtype, significant values are in bold.*

### Surrogate Oncogenes are Predictive of Drug Sensitivity in Breast Cancer Cell Lines

**FIGURE 5:**
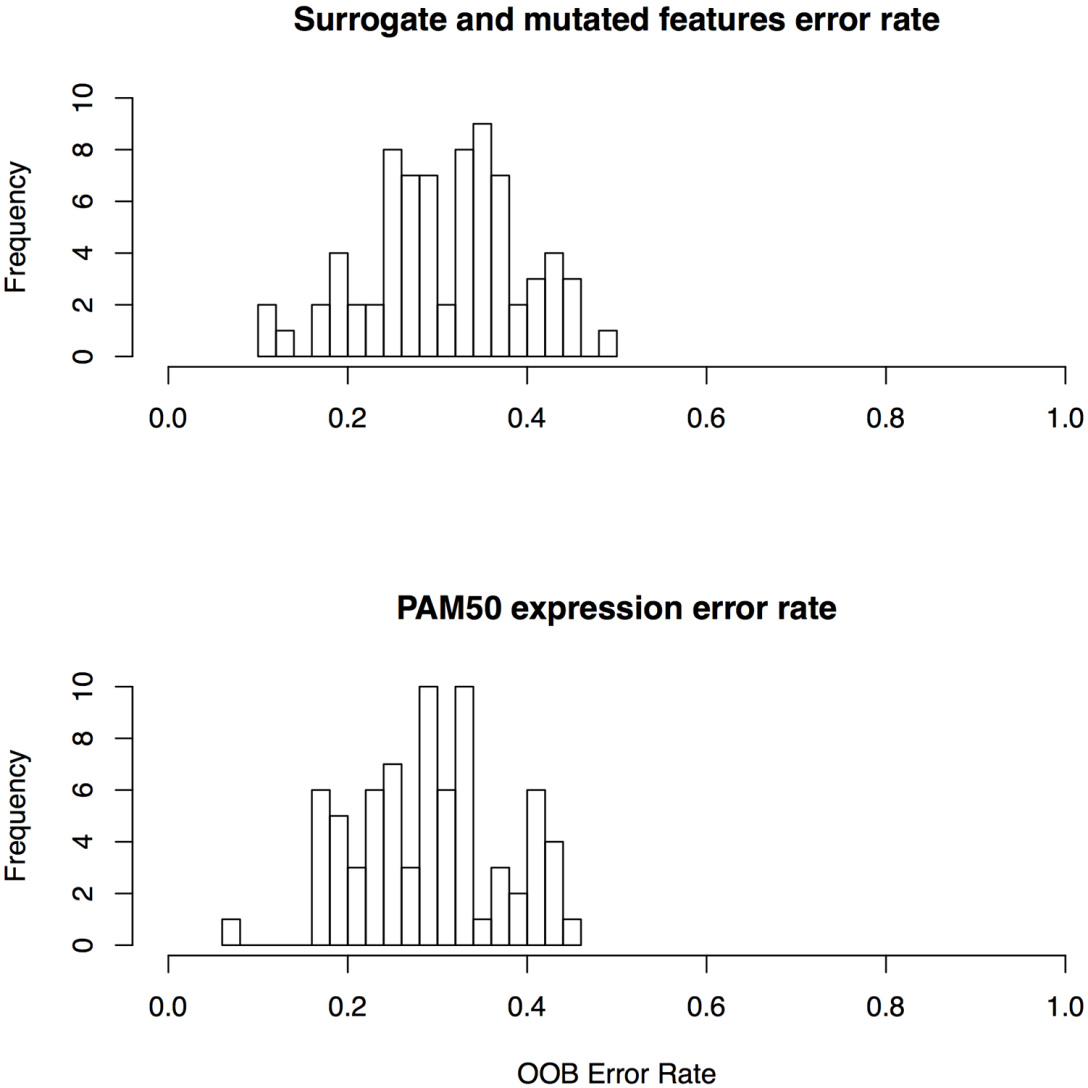
**Error rates for random forest classifiers for surrogate features and PAM50 features**.

We report overall mean OOB error rate over all 72 drugs in our study to assess the performance of drug sensitivity predictors based on surrogate oncogenes. OOB error is the Random Forest equivalent of cross validation error, a measure of generalizability of features used in the classifier. Using surrogate oncogenes as features alone in our RF classifier, we achieve a mean OOB error of 32.1% in predicting whether a cell line is sensitive or insensitive to a drug. In contrast, predictors based on gene mutations yield predictors with a mean OOB error of 43.5%. Overall, the mean OOB error for the combined surrogate/mutated features was nearly identical to the PAM50 expression features (30.9% versus 29.1%, respectively) across all 72 drugs (**Supplementary Table S5**; **Figure 5**). These results suggest that the generalizability of both the PAM50 expression and the surrogate features are roughly equivalent. We also generated a predictor based on linear combinations of PAM50 and surrogate gene RF models, but no combinations yielded improved performance.

Surrogate oncogenes as features consistently predict cell line sensitivity better than the PAM50 features for a number of drugs. In particular, they consistently predict drug sensitivity better for the platinum-based drugs (Cisplatin, Oxaliplatin, and Carboplatin). This may be due to the fact that these drugs affect a large number of targets, suggesting that network-based features may be more predictive than single oncogenic features.

### Surrogates are Associated with Patient Survival

**FIGURE 6:**
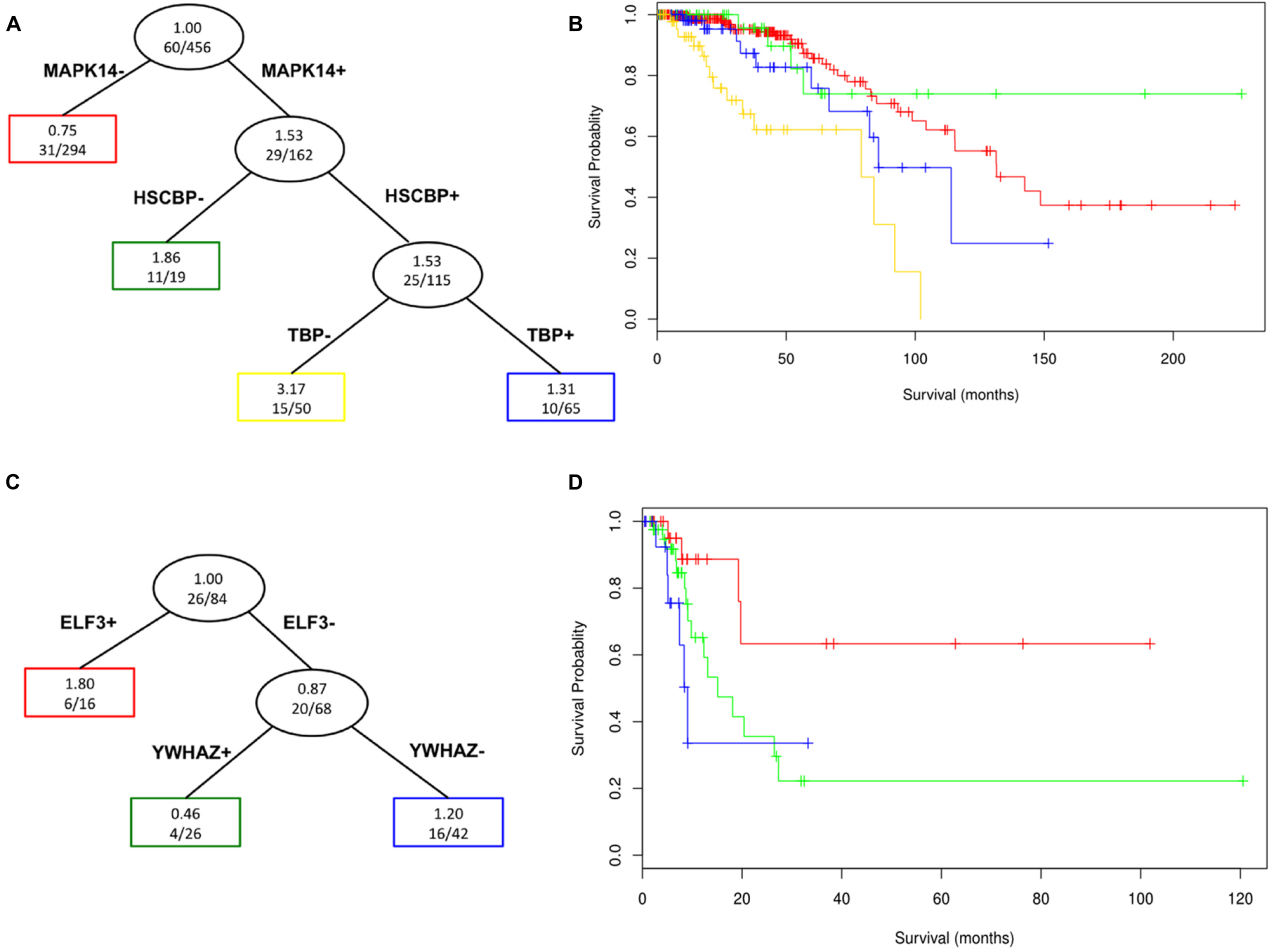
**Survival Tree analysis for surrogate oncogenes in TCGA breast cancer patient data (A and B, n = 456) and TCGA bladder cancer (C and D, *n* = 84) patients of the various nodes (groupings) defined by the survival trees (A and C) are color coded in the corresponding survival plot.** The red group, which does not have a MAPK14 or a HSPCB surrogate, has noticeably longer survival (Log-rank *p*-value = 9.79 x 10-8) than those with these two (blue and yellow) surrogate oncogenes for breast cancer patients. For bladder patients, ELF3+ patients show noticeably lower survival than ELF3- patients (*p*-value 0.0281, *n* = 26), with ELF3+/YWHAZ+ patients having overall lower survival than ELF3+/YWHAZ- patients.

We performed a survival tree analysis to find prognostic features associated with overall survival in the TCGA breast cancer and bladder cancer cohorts. This is highlighted to show the utility of approach for potential clinical applications. For the breast cancer data, there were significant differences in survival based on the surrogate oncogenes (Log-rank *p*-value = 9.79 × 10^-8^; Kaplan–Meier survival curve **Figure 6A,B**). Having both MAPK14 and HSPBC (HSP90) surrogate oncogenes tends to be associated with shorter survival compared to having only MAPK14 mutations. Interestingly, we noted that the MAPK14+/HSPBC– group (green), appears to have overall longer survival than the MAPK14–/HSPBC– group. Within bladder cancer patients, ELF3+ surrogate status is significantly predictive of lower survival (*p*-value 0.0281, *n* = 26, **Figure 6A,B**). We note that limited survival annotation impacts the sample size and so these results will need to be validated in future studies with larger cohorts and more complete follow-up data.

**FIGURE 7:**
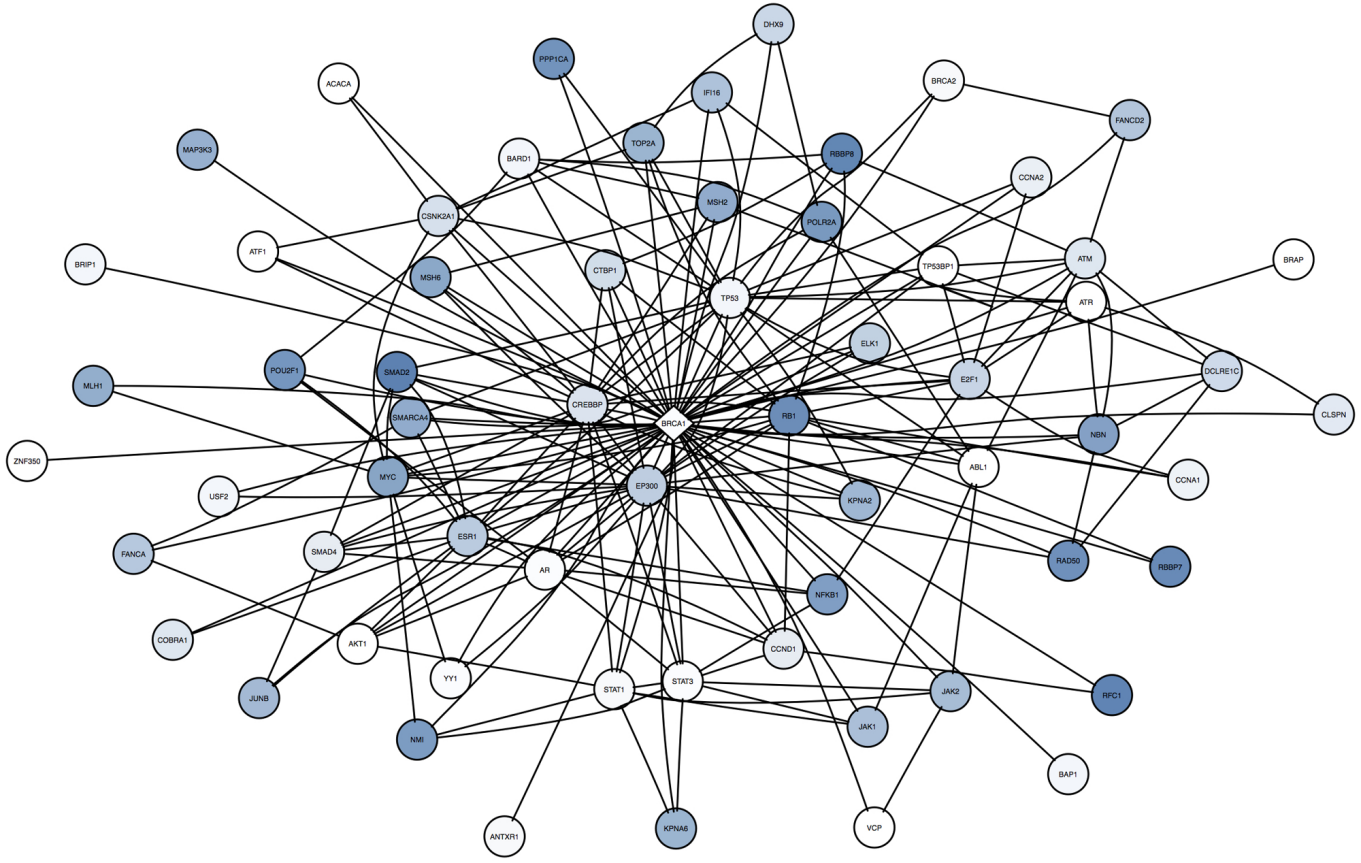
**Across cell line comparison of BRCA1 in cell lines shows unique and rare mutations are incorporated in the analysis.** The center diamond node is BRCA1, the surrogate oncogene of interest. Neighbor nodes are all mutated, and coloring reflects the frequency of occurrence (dark blue = high frequency, white = rare or unique) of that mutated neighbor across all samples. Note that many of the infrequent mutations and alterations (such as STAT1, STAT3, ABL1, and AR are highly interconnected with other mutations in this network, suggesting these infrequent mutations may be highly influential to BRCA1.

## Discussion and Conclusion

Surrogate oncogenes are a new model of oncogenic collaboration, where mutations in proteins that directly interact with oncogenes may affect the function or regulation of oncogenes. This model incorporates rare or unique mutations in an individual sample. By aggregating these neighboring mutations into genomic features we term surrogate oncogenes, we established a network-based background model to filter on statistical significance. We show that surrogate features are predictive of drug sensitivity in breast cancer cell lines. Finally, we show that these surrogate features are prevalent in a number of cancer types in TCGA, including breast, bladder, and GBM.

Many cancers lack highly prevalent driver mutations that can be targeted therapeutically. In this paper, we have shown that surrogate oncogenes incorporate rare or unique mutations not present in larger populations. Such mutations are often disregarded as “passenger” or unimportant mutations that do not carry information. By incorporating their contribution to a surrogate oncogene, we potentially gain more predictive power associated with drug sensitivity and outcome. For example, for individual patients, the inclusion of rare or unique mutations into the surrogate calculation may make a surrogate oncogene significant, implicating it as a possible feature to be associated with drug sensitivity or survival.

We have shown that surrogate oncogenes are useful predictors of drug sensitivity, on par with PAM50 subtype, which is in line with previous studies (Heiser et al., 2012). This is a surprising result, given that surrogate features are categorical and that the expression features used in the PAM50 classifier are continuous expression values. There are at least two reasons why combining the PAM50 and surrogate mutation predictions does not increase our prediction accuracy. First, there is a high correlation between copy number status and expression data, so the two predictors contain redundant information. Second, we may be reaching the upper limits of the prediction in terms of the drug sensitivity problem for the cell lines, so there is little benefit to combining PAM50 and surrogate oncogene information.

However, subtypes derived from NBS, a related technique, also are highly predictive of survival, suggesting that aggregating mutations by their network influence provides information not present in mutations in the oncogenes themselves (Hofree et al., 2013).

The prediction of drug sensitivity, in general, is complicated by the GI50 distributions. Many of the drugs are not evenly distributed across the sensitivity spectrum. For example, 22 of the 30 cell lines have very low GI50 values for methotrexate, making the high and low bins uneven for this compunds. Eleven of the drugs show a left-sided, or negative skewness, and 13 of the drugs show right-sided or positive skewness. Such uneven bins bias the RF predictor towards the larger bin, thereby potentially throwing off the accuracy of the predictor. Based on this factor, we decided to bin the drug response data into even groups of sensitive and resistant samples.

Although there are many cases of alterations and mutations in a target that give rise to drug sensitivity, such as HER2 amplification for HER2 inhibitors such as lapatinib (Bedard and Piccart-Gebhart, 2008; Chakrabarty et al., 2010; Chapman et al., 2011; da Cunha Santos et al., 2011; Baselga et al., 2012; Zecchin et al., 2013), there are many cases of targeted drug sensitivity that do not map to mutations and alterations in the drug target (Garnett et al., 2012). Using frequency-based methods to find such one-to-one gene/drug associations is complicated by the long tail of mutations and alterations (Garnett et al., 2012). To some extent, these cases may be driven by unique and rare mutations in interacting proteins that confer sensitivity to the target. Surrogate oncogene analysis allows for the incorporation of such rare and unique mutations into the interpretation of drug sensitivity.

A cell line with a surrogate oncogene in a druggable target does not necessarily show sensitivity toward that target. To some degree this is expected, as single surrogate oncogenes do not represent the entire network of proteins that may affect drug sensitivity. Instead, it is clear that a combination of surrogates is predictive of drug sensitivity and may be more representative of the influential network.

Using survival analysis, we show that surrogate features are predictive of survival in the TCGA breast and bladder patients, which indicates the potential clinical utility of surrogate oncogene gene sets for patients. Additionally, we have shown that surrogates are associated with molecular subtype in both the cell line and patient data. This suggests that the aggregation of genomic features into surrogate oncogenes captures additional biology behind these molecular subtypes, and should be further investigated in future studies.

One complication of our analysis is that surrogates themselves represent nested and dependent entities. As expected of oncogenes, which tend to be highly connected in the protein-protein interaction network, a large number of mutations for one surrogate oncogene may participate in another surrogate oncogene. The appropriate adjustment for multiple comparisons under these type of nested dependencies is unclear and requires future methodological evaluation. A current method for adjustment under nested dependencies-that of Benjamini–Yekutieli–is not appropriate, as the dependencies are different for each surrogate oncogene (Benjamini and Yekutieli, 2001). Because of the possibility of false positives, experimental validation of survival and drug sensitivity using drug screening assays is needed to validate findings from this approach.

Despite this complication, our findings indicate that surrogate oncogenes can act as a model of continuous haploinsufficiency. Berger et al. (2011) suggest that the two-hit model of recessive tumor suppressor genes (TSGs) should be considered a continuum, influenced by the expression level of the TSGs. Our surrogate model suggests one such mechanism for the regulation of expression levels in TSGs, in that mutations in interacting proteins may affect the regulation and function of proteins.

In summary, by aggregating mutation and copy number data onto PPI networks, we have shown the prevalence of a new type of genomic feature, the surrogate oncogene. Surrogate oncogenes incorporate oncogenic collaboration of rare and infrequently altered genes by summarizing their influence at the oncogene level. Surrogate oncogenes are associated with molecular subtype and are predictive of survival in patients and drug sensitivity in cell lines.

## Author Contributions

TL, LH, and KS jointly conceived of the study. TL devised the statistical framework, R Package, and surrogate oncogene explorer, and evaluated the surrogate oncogenes as biomarkers for drug sensitivity and survival with the TCGA data. LH gave guidance with the breast cancer cell line data and DREAM drug sensitivity data and provided additional feedback on manuscript. KS gave guidance on TCGA data and on manuscript.

## Conflict of Interest Statement

The authors declare that the research was conducted in the absence of any commercial or financial relationships that could be construed as a potential conflict of interest.

## Footnotes

1 http://cbio.mskcc.org/cancergenomics/pancan_tcga/

2 https://www.synapse.org.

3 https://tladeras.shinyapps.io/surrogateShiny/.

## Supplementary Material

**TABLE S1 | Surrogate oncogenes for TCGA Bladder Cancer (BLCA) Patients.** “Gene” indicates surrogate oncogene of interest, “Sample” indicates TCGA patient id in cohort, “neighbor” indicates number of mutated or CNA neighbors (including whether the surrogate oncogene itself was mutated), “degree” is the true number of interactions for the surrogate oncogene in HPRD, “*p*-value” is the pvalue called by the method, and “isMutated” indicates whether the surrogate oncogene itself was altered (1) or not (0). **TABLE S2 | Surrogate oncogenes for TCGA Breast Cancer (BRCA) Patients.** Column titles are identical to **Supplementary Table S1**. **TABLE S3 | Surrogate oncogenes for TCGA Glioblastoma (GBM) Patients.** Column titles are identical to **Supplementary Table S1**. **TABLE S4 | Fisher’s Test of Association of surrogate oncogene status with PAM50 subtype for TCGA BRCA patients.** Test was conducted identically to **Table 2** in main text. **TABLE S5 | Error Rates in prediction of drug sensitivity status using random forests using Surrogate Features versus PAM50 expression.** The better set of features is called for each drug, and the gene target information, if known, is shown.

## References

[B1] Baselga J. Bradbury I. Eidtmann H. Di Cosimo S. de Azambuja E. Aura C. (2012). Lapatinib with trastuzumab for HER2-positive early breast cancer (NeoALTTO): a randomised, open-label, multicentre, phase 3 trial. *Lancet* 379 633–640. 10.1016/S0140-6736(11)61847-3 22257673 PMC5705192

[B2] Bashashati A. Haffari G. Ding J. Ha G. Lui K. Rosner J. (2012). DriverNet: uncovering the impact of somatic driver mutations on transcriptional networks in cancer. *Genome Biol.* 13 R124 10.1186/gb-2012-13-12-r124 PMC4056374 23383675

[B3] Bedard P. L. Piccart-Gebhart M. J. (2008). Current paradigms for the use of HER2-targeted therapy in early-stage breast cancer. *Clin. Breast Cancer* 8(Suppl. 4), S157–S165. 10.3816/CBC.2008.s.012 19158036

[B4] Benjamini Y. Yekutieli D. (2001). The control of the false discovery rate in multiple testing under dependency. *Ann. Stat.* 29 1165–1188. 10.1214/aos/1013699998

[B5] Berger A. H. Knudson A. G. Pandolfi P. P. (2011). A continuum model for tumour suppression. *Nature* 476 163–169. 10.1038/nature10275 21833082 PMC3206311

[B6] Breiman L. (2001). Random forests. *Mach. Learn.* 45 5–32. 10.1023/A:1010933404324

[B7] Cancer Genome Atlas Network (2012). Comprehensive molecular portraits of human breast tumours. *Nature* 490 61–70. 10.1038/nature11412 23000897 PMC3465532

[B8] Chakrabarty A. Rexer B. N. Wang S. E. Cook R. S. Engelman J. A. Arteaga C. L. (2010). H1047R phosphatidylinositol 3-kinase mutant enhances HER2-mediated transformation by heregulin production and activation of HER3. *Oncogene* 29 5193–5203. 10.1038/onc.2010.257 20581867 PMC2945381

[B9] Chapman P. B. Hauschild A. Robert C. Haanen J. B. Ascierto P. Larkin J. (2011). Improved survival with vemurafenib in melanoma with BRAF V600E mutation. *N. Engl. J. Med.* 364 2507–2516. 10.1056/NEJMoa1103782 21639808 PMC3549296

[B10] Ciriello G. Cerami E. Aksoy B. A. Sander C. Schultz N. (2013). Using MEMo to discover mutual exclusivity modules in cancer. *Curr. Protoc. Bioinform. ed. Board Andreas Baxevanis Al. Chap.* 8 Unit 8.17 10.1002/0471250953.bi0817s41 PMC5563973 23504936

[B11] Ciriello G. Cerami E. G. Sander C. Schultz N. (2011). Mutual exclusivity analysis identifies oncogenic network modules. *Genome Res.* 22 398–406. 10.1101/gr.125567.111 21908773 PMC3266046

[B12] Costello J. C. Heiser L. M. Georgii E. Gönen M. Menden M. P. Wang N. J. (2014). A community effort to assess and improve drug sensitivity prediction algorithms. *Nat. Biotechnol.* 32 1202–1212. 10.1038/nbt.2877 24880487 PMC4547623

[B13] da Cunha Santos G. Shepherd F. A. Tsao M. S. (2011). EGFR mutations and lung cancer. *Annu. Rev. Pathol. Mech. Dis.* 6 49–69. 10.1146/annurev-pathol-011110-130206 20887192

[B14] Daemen A. Griffith O. L. Heiser L. M. Wang N. J. Enache O. M. Sanborn Z. (2013). Modeling precision treatment of breast cancer. *Genome Biol.* 14 R110 10.1186/gb-2013-14-10-r110 PMC3937590 24176112

[B15] Dees N. D. Zhang Q. Kandoth C. Wendl M. C. Schierding W. Koboldt D. C. (2012). MuSiC: identifying mutational significance in cancer genomes. *Genome Res.* 22 1589–1598. 10.1101/gr.134635.111 22759861 PMC3409272

[B16] Garnett M. J. Edelman E. J. Heidorn S. J. Greenman C. D. Dastur A. Lau K. W. (2012). Systematic identification of genomic markers of drug sensitivity in cancer cells. *Nature* 483 570–575. 10.1038/nature11005 22460902 PMC3349233

[B17] Gulati S. Cheng T. M. K. Bates P. A. (2013). Cancer networks and beyond: Interpreting mutations using the human interactome and protein structure. *Semin. Cancer Biol.* 23 219–26. 10.1016/j.semcancer.2013.05.002 23680723

[B18] Hanahan D. Weinberg R. A. (2000). The hallmarks of cancer. *Cell* 100 57–70. 10647931 10.1016/s0092-8674(00)81683-9

[B19] Hanahan D. Weinberg R. A. (2011). Hallmarks of cancer: the next generation. *Cell* 144 646–674. 10.1016/j.cell.2011.02.013 21376230

[B20] Heiser L. M. Sadanandam A. Kuo W.-L. Benz S. C. Goldstein T. C. Ng S. (2012). Subtype and pathway specific responses to anticancer compounds in breast cancer. *Proc. Natl. Acad. Sci. U.S.A.* 109 2724–2729. 10.1073/pnas.1018854108 22003129 PMC3286973

[B21] Hofree M. Shen J. P. Carter H. Gross A. Ideker T. (2013). Network-based stratification of tumor mutations. *Nat. Methods* 10 1108–1115. 10.1038/nmeth.2651 24037242 PMC3866081

[B22] Lawrence M. S. Stojanov P. Polak P. Kryukov G. V. Cibulskis K. Sivachenko A. (2013). Mutational heterogeneity in cancer and the search for new cancer-associated genes. *Nature* 499 214–218. 10.1038/nature12213 23770567 PMC3919509

[B23] Looking Across Many Cancer Genomes (2013). *Cancer Genome Atlas – National Cancer Institute*. Available at: http://cancergenome.nih.gov/newsevents/newsannouncements/TCGA_Pan-Cancer_Press_Release_2013 [accessed March 20 2015].

[B24] Mermel C. H. Schumacher S. E. Hill B. Meyerson M. L. Beroukhim R. Getz G. (2011). GISTIC2.0 facilitates sensitive and confident localization of the targets of focal somatic copy-number alteration in human cancers. *Genome Biol.* 12 R41 10.1186/gb-2011-12-4-r41 PMC3218867 21527027

[B25] Mutation Annotation Format (MAF) Specification (2014). Available at: https://wiki.nci.nih.gov/display/TCGA/Mutation+Annotation+Format+(MAF)+Specification [accessed March 11 2015].

[B26] Neve R. M. Chin K. Fridlyand J. Yeh J. Baehner F. L. Fevr T. (2006). A collection of breast cancer cell lines for the study of functionally distinct cancer subtypes. *Cancer Cell* 10 515–527. 10.1016/j.ccr.2006.10.008 17157791 PMC2730521

[B27] Parker J. S. Mullins M. Cheang M. C. U. Leung S. Voduc D. Vickery T. (2009). Supervised risk predictor of breast cancer based on intrinsic subtypes. *J. Clin. Oncol. Off. J. Am. Soc. Clin. Oncol.* 27 1160–1167. 10.1200/JCO.2008.18.1370 PMC2667820 19204204

[B28] Subramanian A. Tamayo P. Mootha V. K. Mukherjee S. Ebert B. L. Gillette M. A. (2005). Gene set enrichment analysis: A knowledge-based approach for interpreting genome-wide expression profiles. *Proc. Natl. Acad. Sci. U.S.A.* 102 15545–15550. 10.1073/pnas.0506580102 16199517 PMC1239896

[B29] Vandin F. Clay P. Upfal E. Raphael B. J. (2012). “Discovery of mutated subnetworks associated with clinical data in cancer,” in *Proceedings of the Pacific Symposium on Biocomputing*, Kohala, HI. 22174262

[B30] Zecchin D. Boscaro V. Medico E. Barault L. Martini M. Arena S. (2013). BRAF V600E is a determinant of sensitivity to proteasome inhibitors. *Mol. Cancer Ther.* 12 2950–2961. 10.1158/1535-7163.MCT-13-0243 24107445

