# Supplementary material for: A Network-Based Model of Oncogenic Collaboration for Prediction of Drug Sensitivity
Source: Front Genet. 2015 Dec 23;6:341. doi: 10.3389/fgene.2015.00341 (PMC4688377; doi:10.3389/fgene.2015.00341)
Supplement: Supplementary file 2 [file Image_1.PDF]

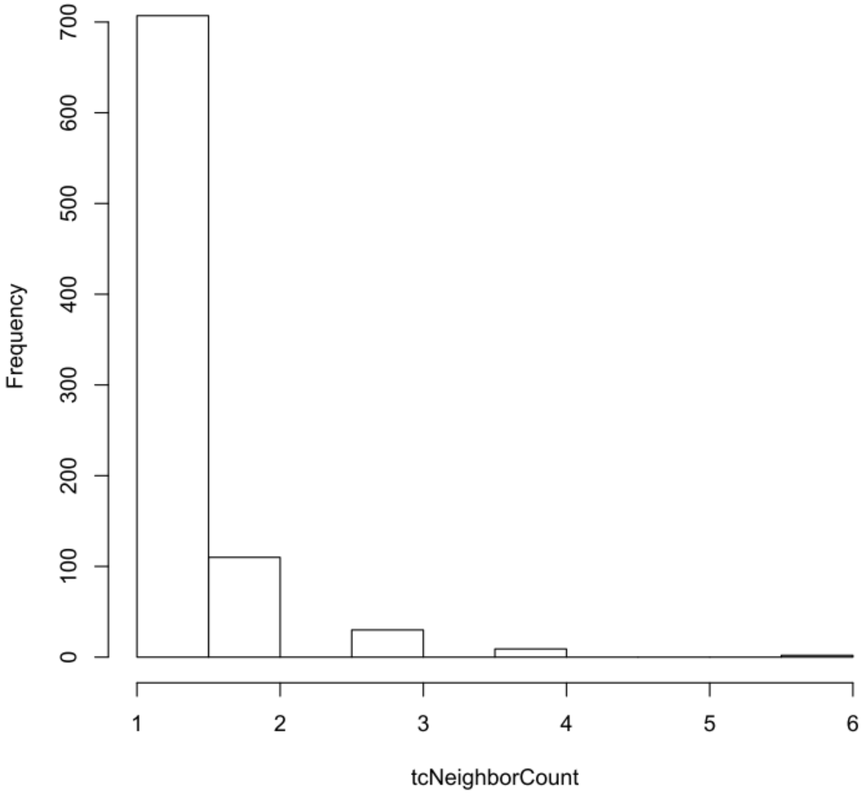

Supplementary Figure 1. Distribution of connections of in-bound connections to core set for Breast cancer surrogate oncogene set. Based on this histogram, an inclusion criteria of 2 connections or more was used to include genes into the surrogate set. Similar distributions were plotted for each core set for each cancer.

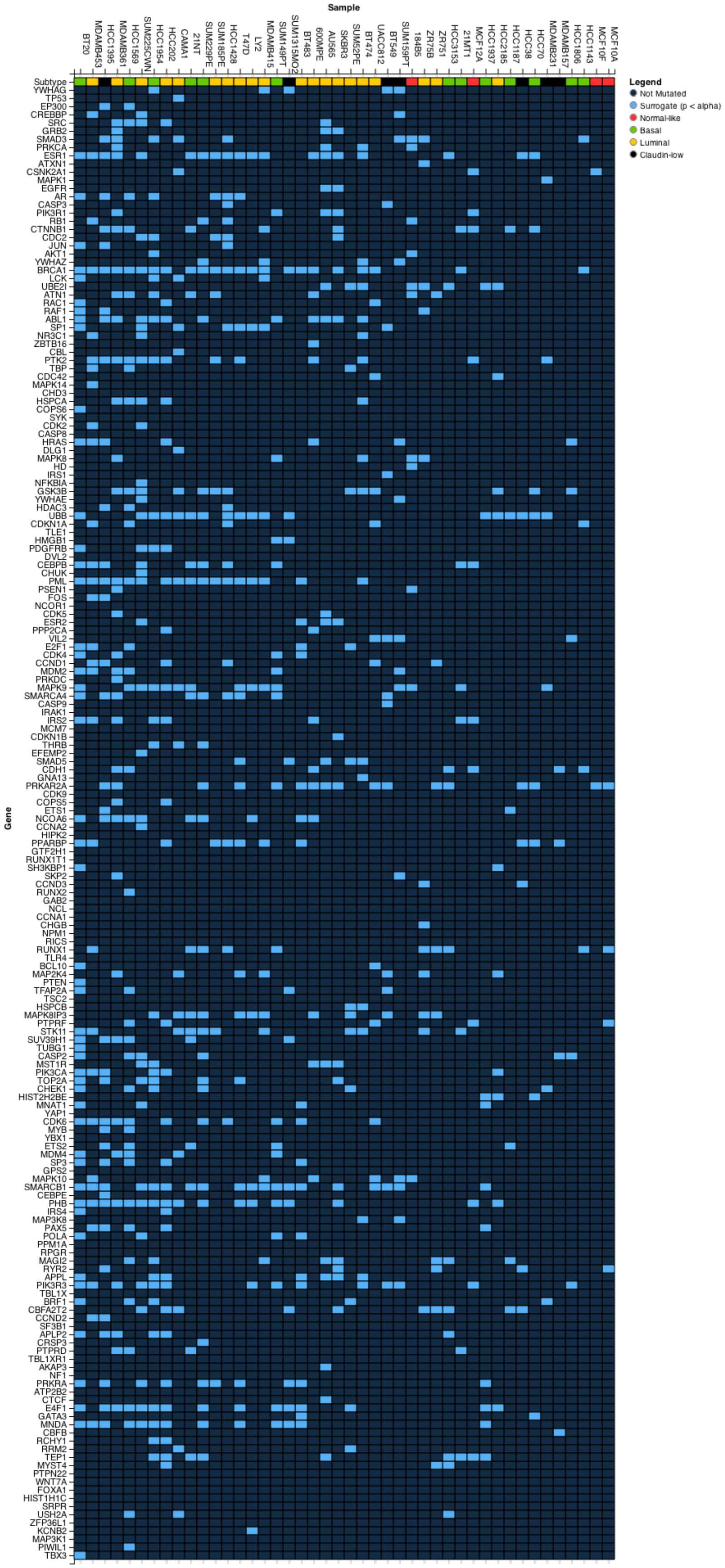

Supplementary Figure 2. Surrogate mutations row-sorted by total number of PPI interactions/surrogate. The number of surrogate mutations for a gene across cell lines is not correlated with total number of PPI interactions for that gene.

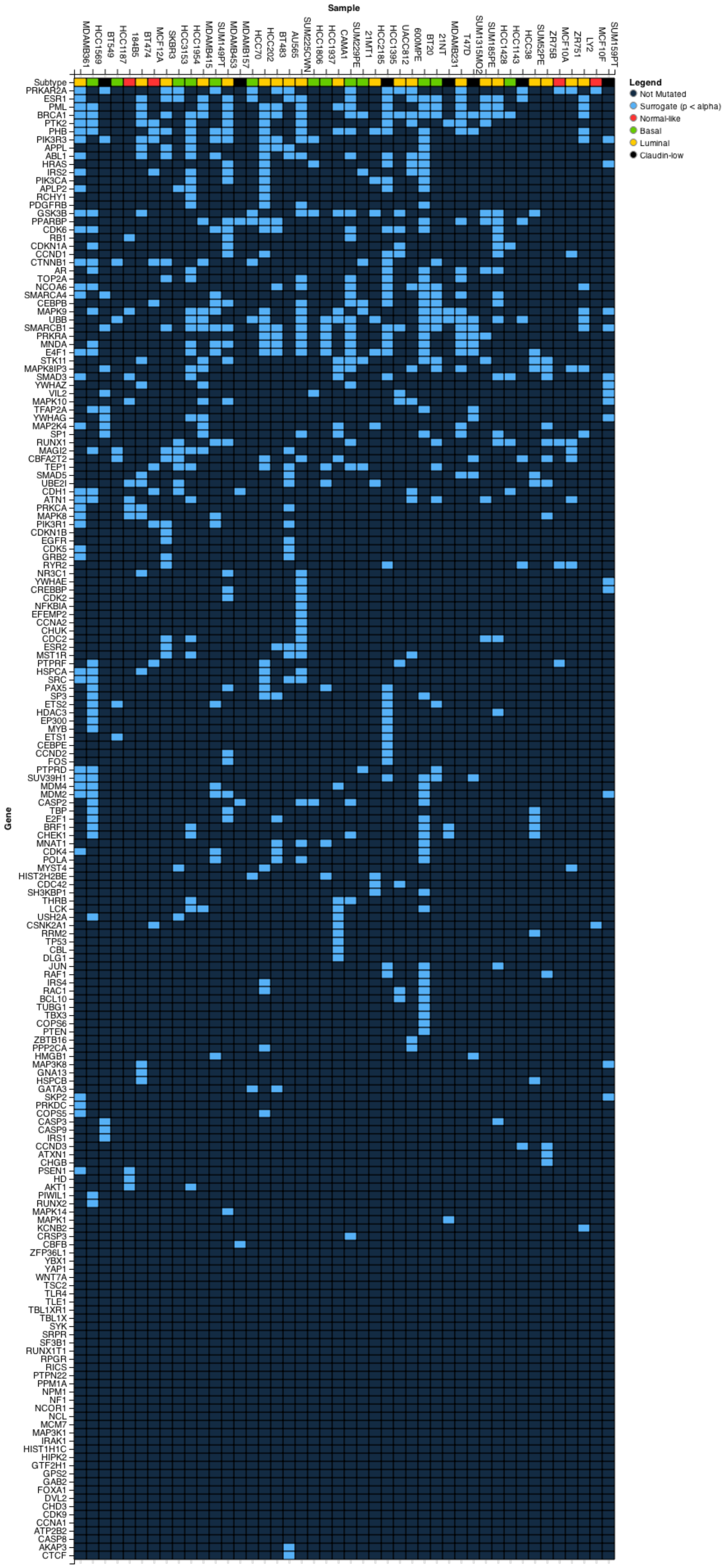

Supplementary Figure 3. Surrogate Mutations column-sorted by total number of observed genomic alterations in a cell line (MDAM361 has the most, SUM159PT has the least). Number of Surrogate mutations is not correlated with total number of alterations.
